# The design and implementation of the re-vitalised integrated disease surveillance and response (IDSR) in Uganda, 2013–2016

**DOI:** 10.1186/s12889-018-5755-4

**Published:** 2018-07-13

**Authors:** Christine Kihembo, Ben Masiira, Lydia Nakiire, Edson Katushabe, Nasan Natseri, Immaculate Nabukenya, Innocent Komakech, Charles Lukoya Okot, Francis Adatu, Issa Makumbi, Miriam Nanyunja, Solomon Fisseha Woldetsadik, Patrick Tusiime, Peter Nsubuga, Ibrahima Soce Fall, Alemu Wondimagegnehu

**Affiliations:** 1 Epidemiology and Surveillance Division, Ministry of Health, P.O BOX 7072 Kampala, Uganda; 2 Public Health Emergency Operations Centre, Ministry of Health, P.O BOX 7072 Kampala, Uganda; 3 World Health Organization, Uganda Country Office, P.O BOX 24578 Kampala, Uganda; 4 National Disease Control, Ministry of Health, P.O BOX 7072 Kampala, Uganda; 5 Global Public Health Solutions LLC, Atlanta, GA 30326 USA; 6 World Health Organization, Africa Regional Office, Brazzaville, Republic of Congo

**Keywords:** IDSR, Training, Multi-disciplinary, Design, Implementation, Uganda

## Abstract

**Background:**

Uganda adopted and has been implementing the Integrated Disease Surveillance (IDSR) strategy since 2000. The goal was to build the country’s capacity to detect, report promptly, and effectively respond to public health emergencies and priorities. The considerable investment into the program startup realised significant IDSR core performance. However, due to un-sustained funding from the mid-2000s onwards, these achievements were undermined. Following the adoption of the revised World Health Organization guidelines on IDSR, the Uganda Ministry of Health (MoH) in collaboration with key partners decided to revitalise IDSR and operationalise the updated IDSR guidelines in 2012.

**Methods:**

Through the review of both published and unpublished national guidelines, reports and other IDSR program records in addition to an interview of key informants, we describe the design and process of IDSR revitalisation in Uganda, 2013–2016. The program aimed to enhance the districts’ capacity to promptly detect, assess and effectively respond to public health emergencies.

**Results:**

Through a cascaded, targeted skill-development training model, 7785 participants were trained in IDSR between 2015 and 2016. Of these, 5489(71%) were facility-based multi-disciplinary health workers, 1107 (14%) comprised the district rapid response teams and 1188 (15%) constituted the district task forces. This training was complemented by other courses for regional teams in addition to the provision of logistics to support IDSR activities. Centrally, IDSR implementation was coordinated and monitored by the MoH’s national task force (NTF) on epidemics and emergencies. The NTF and in close collaboration with the WHO Country Office, mobilised resources from various partners and development initiatives. At regional and district levels, the technical and political leadership were mobilised and engaged in monitoring and overseeing program implementation.

**Conclusion:**

The IDSR re-vitalization in Uganda highlights unique features that can be considered by other countries that would wish to strengthen their IDSR programs. Through a coordinated partner response, the program harnessed resources which primarily were not earmarked for IDSR to strengthen the program nation-wide. Engagement of the local district leadership helped promote ownership, foster accountability and sustainability of the program.

## Background

In 1998, the World Health Organization Africa region (WHO-AFRO) introduced the Integrated Disease Surveillance and Response (IDSR) strategy as one of the approaches to control the disproportionate communicable disease burden in the region [1, 2]. The strategy aimed at strengthening integrated, action-oriented public health surveillance and response at all levels of the health system [3, 4]. In a functional system, all the six IDSR core activities (detection, registration, confirmation, reporting, data analysis and provision of feedback) must operate optimally for prompt public health action [5, 6]. These are enabled by managerial and support functions such as communication, training, supervision, and resource-provision. Since the introduction of IDSR, 46 member states in WHO-AFRO region have adopted and implemented the strategy to varying levels [7]. Although remarkable improvements in surveillance have been realised in some countries, challenges in IDSR core and support functions still prevail in many of them [8–13]. The strategy, however, has achieved positive outcomes in countries where IDSR support functions such as training, supervision and resource provision were optimal [14]. For example, the number of IDSR trained personnel was found to be directly proportional to improvements in IDSR core function indicators in several WHO-AFRO states [15].

Uganda, like many countries in WHO-AFRO region, is disproportionately affected by endemic, emerging and re-emerging communicable disease burden [16–23]. The rapid population expansion, increased human ecosystem interaction and climate change make the country vulnerable to disease outbreaks [24]. It is imperative that Uganda has a robust public health surveillance and response system that can effectively detect, respond to these public health threats and mitigate their impact [25].

Uganda adopted and has been implementing IDSR since 2000 in a phased manner. Implementation commenced with a baseline assessment of the vertical surveillance programs in the country then a 5-year strategic plan and consequent annual work-plans followed. These aimed at creating a functional early warning surveillance system and appropriate public health response [10]. Subsequently, several initiatives were undertaken to strengthen IDSR in Uganda over the years resulting in evident improvements such as early detection and prompt response to outbreaks [26]. The 2009 assessment of the national public health surveillance systems in the country confirmed these achievements and affirmed that the solid IDSR foundation established could be harnessed to build the WHO/International Health Regulations(IHR 2005) core capacities [27, 28]. This assessment, however, uncovered several challenges affecting IDSR implementation including inconsistent and inadequate support for training, support supervision, communication and feedback. The majority of the districts assessed lacked personnel trained in IDSR. Key reporting tools and reference materials were lacking at the operational level [26, 27].

**Fig. 1:**
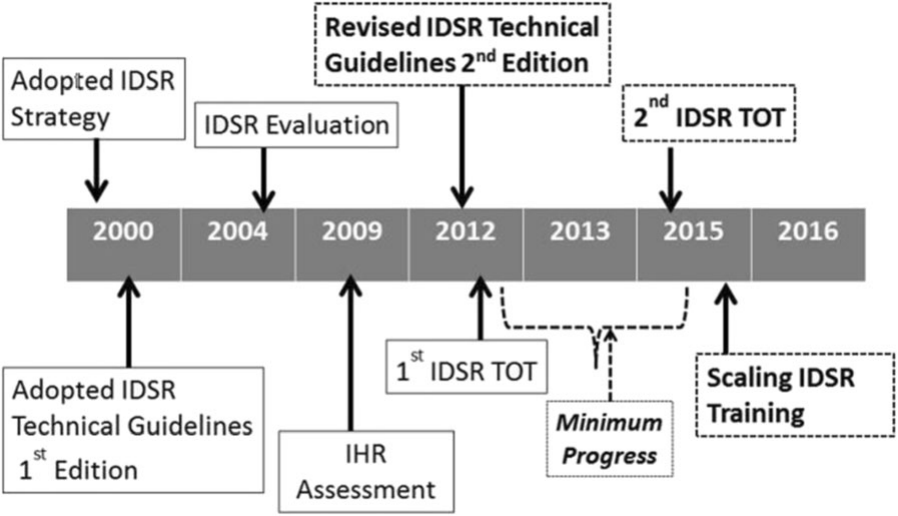
Timeline of implementation of activities to strengthen the IDSR program in Uganda

Following the revision of the generic WHO-IDSR guidelines in 2010, Uganda adopted the changes in revised national IDSR guidelines in 2011. The Ministry of Health (MOH) in collaboration with key partners decided to revitalise the IDSR program and implement the revised guidelines in 2012 (Fig. 1).

The re-vitalisation aimed to enhance the capacity of districts to promptly detect, assess and effectively respond to public health emergencies.

We describe the design and process of IDSR re-vitalisation in Uganda highlighting the roll-out of the revised IDSR guidelines through structured training of the health workforce up to the operational level nation-wide.

## Methods

### Study setting

The Uganda MOH is mandated to lead health service delivery including public health emergencies (PHEs), supported by line sectors such as the agriculture and animal industries ministry in the case of zoonotic disease outbreaks [29]. The national task force (NTF) on epidemics and emergencies is a structure under MOH that coordinates public health emergency response and also is a platform for resource mobilisation, stakeholder engagement and coordination. The NTF reports to the multispectral National Disaster Risk Reduction platform, which platorm ultimately reports to the state cabinet. At the district level, a similar structure, the district task force (DTF) which is an entity of the multisectoral District Disaster Management Technical Committee takes the lead in response to PHEs. Technically, the DTF reports to the NTF [30].

Health service delivery in Uganda is decentralised to districts and health sub-districts (HSD) [31]. By 2015, there were 112 districts within 14 health regions [32]. The health system is structured into national referral hospitals (NRH) at the top, followed by regional referral hospitals (RRHs). These facilities offer a comprehensive health-care including specialised services. General hospitals are at the district level whereas health centre IVs (HCIV) are located at the health sub-districts (HSD) and provide comprehensive services including blood transfusion and comprehensive emergency obstetric care. HCIIIs are found at the sub-county level, are manned by a clinical officer and offer both basic in-patient and out-patient services, whereas HC IIs are at the parish level and provide only out-patient services. The HC I has no physical structure but comprises a village health team(VHT) which links the community to the health system [33]. The district level is the focus for integrating and implementation of IDSR, in addition to health workforce management, support supervision plus health system performance monitoring. The district health department receives reports from HSDs and submits an aggregated report to the national level electronically. The HSD oversees health services within health facilities within the catchment area, receives surveillance reports from these units and submits aggregate data to the district. The District Surveillance Focal Person (DSFP) oversees surveillance activities in the district supervised by the District Health Officer (DHO) [34]. Laboratory or surveillance focal persons at each level ensure safe specimen processing or referral to regional or centralised reference laboratories via the laboratory hub system. Laboratory results are delivered via the reverse mechanism [35].

### Study design and procedures

We reviewed published and unpublished national policy documents and guidelines to document the IDSR implementation framework in Uganda, from 2001 to 2016. We also reviewed preparedness and response protocols developed during the study period, in addition to unpublished reports and minutes of the NTF meeting on epidemics and emergencies. Reports and minutes from the technical working group meetings held for planning and monitoring of the IDSR program during the study period were also reviewed. We reviewed the respective training curriculum documents, training materials, training reports and trainee databases to understand the design, organization and conduct of the training. We also interviewed 18 key informants at the national and district levels to understand the implementation of the training. For information on program funding, we interviewed 10 surveillance focal persons at the districts, MOH and the WHO Country Office(WCO). We also reviewed the program financial documents including budget documents, procurement and equipment handover records to understand logistics and resources deployed in the process.

### The design and development of the nation-wide IDSR training plan

The 2009 IDSR assessment in Uganda revealed that while substantial resources had been allocated for establishing IDSR in the early to mid-2000s, the marked reduction of funds for IDSR systems over the years led to a loss of previously attained capacities particularly at the operational level [26, 27]. Analysis of the country’s responses to disease outbreaks also showed several bottlenecks that needed to be addressed collectively in a coordinated manner. Among them, was a need for a harmonised outbreak response and information flow at the district level.

In 2012, national stakeholders held several NTF meetings to discuss ways of revitalising IDSR in Uganda. They finally reached a consensus about building a competent workforce at the sub-national level to ensure a solid IDSR system. A plan, to implement a nationwide IDSR training to health facilitiesbased on the revised IDSR guidelineswas developed. The training plan was costed at an average cost of US $230 per trainee. (Makumbi, I., Personal communication). The training was planned to be accompanied by systematic follow-up visits, regular monitoring and support supervision to improve reliability and quality of the system gradually in a manner that could ensure sustainability and foster accountability. However, between 2013 and 2014, the implementation of the plan stalled mainly due to lack of funding.

### Risk of Ebola virus importation from West Africa, a catalyst to IDSR revitalisation

The Ebola virus disease (EVD) outbreak in West Africa in 2013–2014 posed an imminent risk of importation of the virus to Uganda owing to the many Ugandans supporting the response in West Africa and the unrestricted international travel and trade. MOH together with partners developed a national EVD/Viral Hemorrhagic Fever (VHF) preparedness and response plan and mobilised resources locally to enhance the country’s readiness. Drawing from the lessons of the EVD outbreak in West Africa, and in line with MOH’s decision in 2012, this plan prioritised training for frontline health workers to strengthen surveillance and response capacities within the IDSR framework. This training was complemented by building capacity in EVD case management, infection prevention and control (IPC), in addition to the regional capacity for laboratory verification and confirmation (Table 1).

**Table 1:** The multi-disciplinary training of health workers conducted to revitalise IDSR in Uganda, 2015–2016

	Type of Training	Level of Training	Target Audience	Duration of Training	Funder
1	IDSR Training	District based	Operational health workers and the District Health Team (DHT) District rapid response teams (DRRT): comprised of DHT and other “one health” technical personnel; District Task Force (DTF): The DRRT plus district technical and political leadership	5 days	UK Aid Department for International Development (DFID) WHO-AFRO, Polio eradication initiative, Central Emergency Response Fund, the Newborn Adolescent and Child Health (RMANCH) continuum of care, and USAID through WHO,
2	Viral Hemorrhagic Fevers (VHF) case management and IPC	Regional	Regional teams comprised of mainly clinicians (doctors, nurses, clinical officers) and non-clinicians (hygienists, ambulance drivers, mortuary attendants, askaris etc).	5 days	DFID, WHO
3	Training for laboratory workers on sample collection, packaging and transportation of infectious specimens	Regional	Regional teams comprised of Laboratory health workers, hub riders, postal service personnel	5 days	United States Centres for Diseases Control and Prevention (CDC)
4	Other Trainings: District level epidemiology training program	District based, modular	District Health Officers, District Veterinary Officers, District Laboratory Focal Person, Health sub-district in-charges	3 month	Defense Threat Reduction Agency (DTRA) through the US CDC

### 1-District based IDSR training for facility-based health workers, district health team (DHT), district rapid response team (DRRT), and the district task force (DTF)

This training aimed at strengthening the functioning and performance of the national disease surveillance system in addition to enhancing capacity and structures for epidemic preparedness and response at the district level [34]. The expected outcomes of the training were; creation of a critical mass of operational health workers competent in IDSR core activities and support functions, the DRRT competent in outbreak investigation and the DTF competent in epidemic preparedness and response.

Cascade training beginning with the training of national-level trainers (TOT), who then conducted district training was done. Districts were trained in phases starting with high-risk districts (disease outbreak-prone, border districts and those with < 60% completeness of the weekly epidemiological reporting in the previous quarter).

**Fig. 2:**
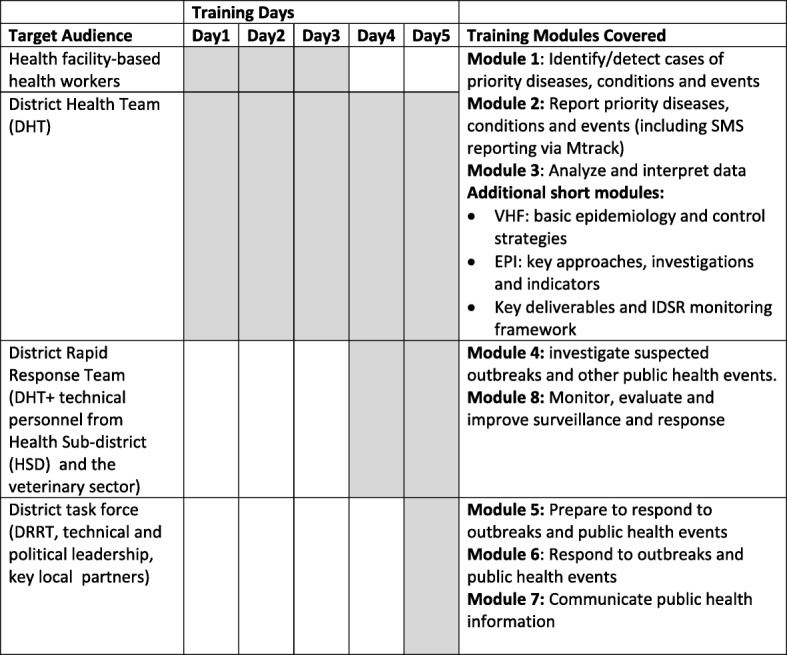
Structure of the IDSR Training in Uganda, demonstrating module selection and mix for competence and skill development

Based on a customised IDSR training manual, the training content was organised into eight modules to cover surveillance and public health action core activities and support functions (Fig. 2).

Participants covered specific modules relevant to their roles and tasks in IDSR with emphasis on relevant skill development. Training in the form of facilitated workshops with guided practice sessions was held at both national and district levels. Case scenarios generated from real-life events were included to facilitate competence and skill acquisition. Efforts were made to integrate different programs. For-example modules on m-Trac (a mobile telephone SMS based information system), electronic Health Management and Information System (HMIS), plus tools for maternal and perinatal deaths surveillance were incorporated into the training [36–38]. Participants who had not used the mTrac system were oriented and registered on the platform.

i) Training of health facility-based health workers: From the IDSR core function matrix, IDSR core activities performed at this level are mainly case detection, reporting and data analysis [5, 39]. While these health workers are expected to participate in other IDSR core activities, they are usually supported by the higher levels. Training of operational health workers targeted 80% coverage of the public sector. These were trained during the first 3 training days, covering modules 1, 2 and 3 (Fig. 2). Additional short modules covered included VHFS’ control overview, key strategies and indicators of the national Expanded Programme on Immunisation (EPI), plus the IDSR monitoring framework.
ii) District rapid response team (DRRT) training: A DRRT is a technical, multi-disciplinary team that is responsible for conducting initial outbreak investigation and response within the district. DRRTs were trained on training day 4 covering modules 4 and 8 (Fig. 2). The target was the DHT, the HSD in-charge and DSFP, plus a few staff selected from high priority health facilities
iii) District task force (DTF) training: The DTF is a coordinating committee composed of technical members from various sectors in addition to the district political and technical leadership. The DTF’s role is to develop and oversee the implementation of emergency preparedness strategies, action plans and procedures [34]. The 1-day DTF training was to enhance district capacity to prepare for and coordinate public health emergency response and this training covered modules 5, 6 and 7(fig. 2). To engage the district leadership to provide oversight of the program, the permanent secretary MOH issued a circular notice to targeted districts. The letter advocated for a functional disease surveillance and early warning system and pointed out expected key deliverables following IDSR training. This letter was delivered by the central facilitators during a courtesy and mobilisation visit to the district leadership through the DHO prior to the DTF training.
iv) Performance monitoring and accountability framework: A framework for monitoring IDSR performance in the district with key deliverables and indicators was collectively agreed upon during the training. The two indicators selected for monitoring IDSR performance were; the proportion of health facilities submitting weekly surveillance report on time to the district level and the proportion of health facilities conducting a detailed case-based investigation for acute flaccid paralysis and measles cases [34]. Also, the DHT/DTF resolved to include the district’s IDSR performance as an agenda item for discussion during all regular health committee meetings. With support from the district leadership, trainers led a detailed discussion of factors that could affect attainment of these indicators and subsequently supported teams to develop tailored action plans to mitigate the issues. Clear next steps and commitments were obtained and followed upon during quarterly support supervision activities and IDSR review meetings.

At the national level, weekly IDSR performance is monitored by both the epidemiology and surveillance/health information units which provide feedback to the districts through a weekly epidemiology bulletin.

Through the WHO country office, funding for the IDSR training was mobilised from various partners and initiatives including the UK-aid Department for International Development (DFID), WHO-AFRO, the continuum of care for Reproductive Maternal Newborn Adolescent and Child Health (RMANCH), USAID, the Global Polio Eradication Initiative (GPEI) and the Central Emergency Response Fund (CERF) (Table 1).

### 2. Regional training on VHF case management and IPC

This training aimed to build a competent regional team of health workers who could comprehensively manage patients with suspected and confirmed VHFs in the case of an outbreak. The training focused on patient management, IPC, establishment and layout isolation facilities, assembling and operations of mobile teams, safe and dignified burial of VHF victims plus VHF preparedness within the IDSR context. With funding from the DFID through WHO, a national TOT was conducted first and cascaded to the regions. The training was based on the national guidelines, and standard operating procedures (SOPS) adapted from the WHO manual for the care and management of patients in Ebola care units [40].

### 3. Regional training for laboratory workers on sample collection, packaging and transportation of infectious samples

To strengthen capacity for laboratory confirmation, laboratory workers were trained on collection and transportation of infectious specimens with support from the US Centres for Disease Control and Prevention under the global health security agenda program. The training aimed at building regional capacity for quality and safe sample referral of infectious specimens within the national laboratory hub network and also targeted staff that interface with specimens via the hub system. The training content included biosafety and biosecurity management principles, bio-risk assessment, IDSR/IHR overview and the role of the laboratory, sample collection and shipment using the International Air Transport Association(IATA) guidelines, the national laboratory hub network operations and laboratory polio control strategies.

### 4. Program coordination, harmonisation and oversight

The coordination of the preparedness and response plan was through the NTF. At MOH, the Director General, Health Services (DGHS) provided technical oversight, supported by technical staff from MOH. The Epidemiology and Surveillance Division, MOH oversaw direct implementation of the program activities with technical support from WHO Country Office (WCO) and other key partners.

## Results

The initial IDSR TOT was held in 2013 and targeted 30 national trainers. However, the majority of these trainers were deployed to West Africa to support the response to EVD outbreak between 2013 and 2014. Another TOT was conducted in 2015, training 26 participants. National trainers were drawn from the WHO Country Office, MOH departments and regional referral hospitals and the academia. The regional EPI /IDSR focal persons were prioritised. The pool of national trainers assisted by district trainers enabled concurrent training of multiple districts. Trainers were also identified from DHTs of well-performing districts (districts with consistent weekly HMIS reporting over the previous quarter) to share their best practices and enriched the training.

### Rapid scale-up of district-based IDSR training

Between 2015 and 2016, 7785 participants were trained in IDSR in all the districts in the country by a pool of national and district trainers. Of the 7785 participants trained, 5489 (71%) were health facility-based health workers, 1107 (14%) were DRRT members, and 1188 (15%) constituted the DTF. Among the DTF participants, representatives of key partners and non-governmental organisations within the districts were included.

Health facility-based participants were from various disciplines, including clinicians (nurses, clinical officers and doctors), laboratory staff, HMIS/records and surveillance officers. Participants were drawn from all levels of the health system; 79% (4337/5489) of the operational health workers were from lower level facilities (HCIIs and HCIIIs), 11% were from HCIVs and 10% from the district and regional referral hospitals. All the districts had more than 80% of the public health facilities represented. High volume private for private health facilities were also included (Table 2).

**Table 2:** The distribution of the operational health workers trained during the IDSR revitalization in Uganda, 2015–2016 by level of health facility

Health Region	Level of Health Facility *Number (%)*				Time of Training *Month/Year*
	Health Centre II	Health Centre III	Health Centre IV	Hospital	
Kampala	418 (46)	288 (32)	90 (10)	115 (13)	Jun- 2016
Mbarara	312 (59)	108 (20)	72 (15)	35 (7)	Feb- 2016
Masaka	149 (39)	140 (37)	59 (15)	35 (9)	Dec-2015
Fort Portal	234 (54)	122 (28)	50 (12)	24 (6)	Nov-2015
Jinja	391 (56)	198 (29)	64 (9)	41 (6)	Nov-2015
Kabale	93 (42)	57 (26)	56 (25)	15 (7)	Nov-2015
Mubende	186 (70)	47 (18)	15 (6)	19 (7)	Nov-2015
Soroti	175 (45)	119 (31)	51 (13)	44 (11)	Nov-2015
Lira	98 (41)	90 (38)	43 (38)	9 (4)	Sep-2015
Gulu	147 (48)	100 (33)	24 (8)	36 (12)	Jul-2015
Mbale	137 (35)	160 (41)	34 (9)	56 (14)	Jun-2015
Arua	107 (29)	194 (53)	34 (9)	33 (9)	May-2015
Hoima	52 (30)	56 (32)	31 (18)	37 (21)	May-2015
Moroto	98 (52)	61 (32)	7 (4)	23 (12)	May-2015
Total	2597 (47)	1740 (32)	630 (11)	522 (10)	

**Fig. 3:**
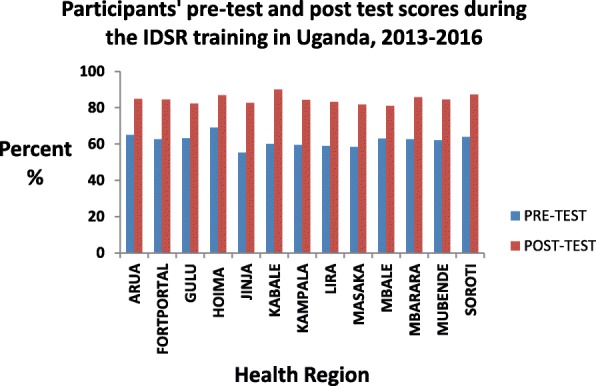
Participants’ pre and post-training assessment scores showing knowledge gain across all health regions during the IDSR training in Uganda 2015–2016

Review of participant’s pre-and post-training assessment scores showed an average knowledge gain of 23%, consistent across all districts (Fig. 3).

In 2015**,** 488 frontline health workers underwent training on VHF case management and infection prevention and control (IPC). Regional teams comprised 337(69%) clinicians including doctors, nurses clinical officers and 134 (28%) non-clinicians such as hygienists, ambulance drivers, mortuary attendants and 17(3.5%) national trainers (Table 3).

**Table 3:** Categories of health workers trained in VHF case management and IPC plus those trained in safe sample collection and referral in Uganda, 2015–2016

Health worker category	Numbers trained *(%)*	Time of Training
Training on VHF case management and infection prevention and control		
Clinicians	337 (69)	Sept-Dec 2015
Non-Clinicians	134 (27.5)	Sept-Dec 2015
National Trainers	17 (3.5)	Jul-2015
Training on sample collection, packaging and transportation of dangerous pathogens for laboratory health workers		
Laboratory workers	208 (91.6)	
Laboratory hub riders/Transporters	4 (1.8)	Sep 2015- Aug 2016
Postal service personnel	15 (6.6)	

Also, 227 participants underwent the laboratory training from eight of the 14 health regions between 2015 and 2016. Participants included 208 (91.8%) laboratory workers, 4(1.8%) hub motorcycle riders and 15(6.6%) postal service personnel. Post-training follow-up and mentorship were provided in an integrated manner. Specifically, within the IDSR/EPI framework, regional focal persons provide technical support supervision to the districts with a special emphasis on IDSR /EPI services on a quarterly basis. Also, by the end of 2016, 30 high-risk districts had had at least one additional support supervision visit from the central team to further consolidate IDSR/HMIS and m-Trac functioning with support from UNICEF.

### Other resources, logistics and initiatives deployed to support IDSR/IHR strengthening.

With support from UNICEF, DFID and WHO-AFRO innovations including m-Trac and e-surveillance were adopted to boost IDSR performance in 2012 [36, 40]. These innovations replaced the traditional cumbersome system for transmitting weekly surveillance and Health Management and Information System (HMIS) data from one health facility level to another physically.

The revised national IDSR/IHR guidelines containing updated surveillance tools and the revised surveillance thresholds booklets were provided as job aides/reference documents to each participant during IDSR Similarly, national guidelines with standard operating procedures (SOPs) on VHF management and IPC were developed to guide response at various levels of the health system. The national curricula for IDSR and VHF case management courses were developed based on the respective guidelines/SOPs (Table 4).

**Table 4:** Some of the resources and materials deployed to support IDSR/IHR strengthening in Uganda, 2013–2016

Resource category	Type of resource	Comments
National curricula, guidelines, Standard Operating Procedures (SOPS) and job aids	− National Technical Guidelines for IDSR, 2nd Edition	The revised guidelines were printed and disseminated to participants (Health facility, DHT and DRRT)
	− National curriculum on IDSR/IHR	National curriculum developed, used for IDSR training
	− Case definitions and epidemic thresholds for IDSR- a working guide for health workers	Working guide updated, printed and provided to participants (Health facility, DHT and DRRT)
	− Guidelines and SOPS for responding to Ebola/Marburg virus outbreaks in Uganda	SOPS and guidelines adapted by a multi-sectoral National Task Force (NTF) team. Printed copies availed at national level
	− Curriculum on VHF case management and infection prevention and control (IPC)	National curriculum adapted and used during VHF case management training
	− Updated vaccine-preventable diseases case investigation forms (Polio/AFP, NNT, Measles, AEFIs)^a^	Updated case investigation forms disseminated during IDSR training
	− VHT handbook on community-based disease surveillance.	The training manual was developed
	− Curriculum on VHF case management and IPC	National curriculum adapted
Contingency medical and laboratory supplies	− Kits and Shipment boxes for laboratory specimens	300 laboratory kits supplied
	− 5 tents each with a 20-bed capacity designed to serve as mobile isolation facilities	Tents were procured and prepositioned
	− Personal protective equipment (PPE) and medical supplies	500 sets of PPEs and assorted medical supplies procured and prepositioned at regional and district hospitals
Other logistical support to strengthen surveillance and IDSR/IHR	− A walk through thermoscanner	A walk-through scanner was procured and installed at the international airport to support screening of incoming travellers.
	− Motorcycles and Vehicles	Motorcycles and vehicles procured and given to high-risk districts to support surveillance activities
MTRACK and Electronic HMIS reporting via DHIS2 System	− A short message mobile telephone based reporting platform for weekly HMIS reporting − Electronic HMIS introduced via the DHIS2 system (open source web-based) introduced	m-trac systems introduced and integrated with the DHIS2 for e-HMIS reporting

^a^*AFP* Acute Flaccid Paralysis, *NNT* Neonatal Tetanus, *AEFIS* Adverse events following immunisation, *HMIS* Health Management and Information System, *DHIS 2* District Health Information Software, *DHT* District Health Team, *DRRT* District Rapid Response Team

Due to the lack of isolation units in the country, MOH with support from DFID through WHO procured five tents, each with a 20-bed capacity designed to serve as emergency isolation facility that can quickly be deployed in case of a VHF outbreak. Assorted drugs, medical supplies and over 2000 sets of personal protective equipment were also procured and pre-positioned at RRHs and district hospitals. The same funding additionally supported the ports of entry(POE) surveillance at the Entebbe International Airport through the acquisition of a walk-through thermal scanner and baseline assessment for IHR capacities at ground POE.

Additionally laboratory supplies including kits and shipment boxes were procured to aid referral of laboratory specimens from districts for prompt diagnosis at the national reference laboratory with financial support from DFID and US Department of Defence. The GPEI and Central Emmergency Response Fund also supported procurement of motorcycles that were provided to strengthen IDSR/EPI surveillance and investigation activities at least 60 high risk districts.

## Discussion

The IDSR revitalisation program in Uganda surpassed the WHO threshold of having trained health workers in IDSR in ≥80% of the districts for effective IDSR performance and achieved nation-wide coverage in 2 years [41]. Significant participant knowledge gain noted across the board was noted across the board, and this was further complemented with post-training support through the integrated support supervision. The number of trained health personnel has been shown to be associated with improvements in IDSR functioning particularly regarding completeness and timeliness of reporting, analysis, interpretation and use of surveillance data at the source [9, 15, 42]. The program also equipped all health workers trained with job aids such as case definitions, surveillance thresholds, reporting forms and national IDSR guidelines. Such tools have been shown to be largely missing at peripheral health system levels in many countries, yet are essential to support health workers in executing day today IDSR activities such as reporting [8, 43, 44].

The design and process of the program implementation also provide valuable and key considerations. Firstly, the IDSR training brought together health workers of multiple disciplines. In higher health facilities, this helped health workers who are primarily not involved in surveillance activities appreciate their indirect yet critical contribution to the program and helped foster teamwork. In lower health units, frontline health workers were identified and equipped with skills to execute relevant IDSR at that level. Training of laboratory staff through the two complementary courses helped supplement the efforts to strengthen laboratory capacity for appropriate pathogen confirmation and sensitivity monitoring, a critical yet one of the weakest program components in the WHO-AFRO region [45–47]. A multi-disciplinary team consisting of the DHT, HSD, and high volume health facilities were trained as the DRRT. This approach was to ensure that competent one health teams that can readily be mobilised and deployed for rapid outbreak investigation and response are available within the districts. Veterinarians at the district level were also included in the DRRT training in the spirit of one health. In contrast to earlier IDSR training where participants covered all eight modules, participants in this training only covered modules that were considered the most crucial to building their required primary skill-set according to the WHO-IDSR function matrix [5]. This approach provided participants adequate time to concretise the content and also shortened the duration participants spent away from duty-stations which is a concern during capacity building initiatives for in-service personnel [15].

Secondly, the cascade approach generated a pool of trainers that enabled concurrent training of multiple districts. Preparations for the training followed a standard checklist, and continuous quality improvement was made based on the end of training evaluation. Districts were engaged to do the selection and mobilisation of participants according to preset criteria. Districts also handled logistical and administrative preparations of the training, an approach that promoted district involvement and ownership of the program. The best practices shared by the district trainers enriched the training and participant action plan development.

Thirdly, the integration of other modules into the IDSR training fostered complementarity of programs, minimising wastage of resources due to duplication of efforts. This was further strengthened by NTF coordination and oversight of the program. Though more elaborate content was covered in the regional VHF and laboratory courses, the training was conducted within the IDSR framework enhancing complementarity.

Fourthly, drawing from lessons learnt during the Ebola outbreak in West Africa, the importance of prompt and effective community engagement in outbreak preparedness and response cannot be underestimated [48–50]. This program approached this component through engagement of the district technical and political leadership to oversee the IDSR implementation and accountability framework. More still, district political leaders in Uganda have free weekly air-time access to advocate, strengthen and monitor government programs through major local media houses in the district. The same platform could be leveraged to further engage and involve the local communities in the IDSR program.

Lastly, the program illustrated how funding from both national programs and partners that otherwise was not directly earmarked for IDSR activities was harnessed to strengthen the program. Lack of sustainable funding of IDSR activities is the major challenge in IDSR implementation in many countries, Uganda inclusive [14]. The program, thus anticipated that funding for IDSR would be incorporated into the districts’ budgets, once the program’s importance and utility were appreciated by the district leadership. More-still, the WHO /IHR and the CDC global health security agenda advocate for countries to allocate a budget line at the central level to facilitate IDSR implementation which is the vehicle to implement IHR in the region [28].

One limitation in this study is that the key informants’ views could have been were influenced by their role in the programme. They could have had a natural bias to focus on positive aspects of the programme and the reviewed records could have forcused on programme successes. However we tried to tease out the pertinent issues and also we triangulated sources of information to mitigate the effect of the limitation.

## Conclusion

The re-vitalisation of the IDSR program in Uganda highlights unique features which can be easily adopted and applied by other countries that would wish to strengthen their IDSR programs. Through a coordinated partner support and response, funding which was not primarily earmarked for IDSR implementation was mobilised and harnessed to achieve nation-wide equipping of multi-disciplinary district teams with skill-sets and tools necessary for performing relevant IDSR functions. The program also promoted engagement of local district leadership which is critical in fostering accountability and is a positive step in exploring sustainability possibility of the program as the long-term funding from governments for global health security is pursued. We demonstrate that when partners work collaboratively and pool their efforts together in a coordinated manner, a significant impact on public health can be achieved countrywide.

## Abbreviations

AFP: Acute Flaccid Paralysis
CERF: Central Emergency Response Fund
DFID: Department for International Development
DGHS: Director General Health Services
DHIS2: Disrict Health Information Software 2
DHO: District Health Officer
DHT: District Health Team
DRRT: District Rapid Response Team
DSFP: District Surveillance Focal Person
DTF: District Task Force
EPI: Expanded Program on immunization
EVD: Ebola Virus Disease
GPEI: Global Polio Eradication Initiative
HC: Health Centre
HMIS: Health Management and Information System
HSD: Health Sub-district
IATA: International Air Transport Association
IDSR: Integrated Disease Surveillance and Response
IHR: International Health Regulations
IPC: Infection Prevention and Control
MOH: Ministry of Health
NTF: National Task Force on Epidemics and Public HealthEmergencies
PHE: Public Health Emergencies
POE: Port of Entry
RMANCH: for Reproductive Maternal Newborn Adolescent and Child Health
RRH: Regional Referral Hospital
SOPS: Standard Operating Procedures
TOT: Training of Trainers
UK: United Kingdom
UNICEF: United Nations Children’s Fund
US CDC: United States Centers for Disease Control and Prevention
USAID: United States Agency for International Development
VHF: Viral Hemorrhagic Fever
VHT: Village Health Team
WCO: World Health Organization Country Office
WHO: World Health Organization
WHO-AFRO: World Health Organization Africa Regional Office
